# Targeting tumour-infiltrating B cells: mechanisms and advances in cancer therapy

**DOI:** 10.1038/s41419-025-08254-z

**Published:** 2025-11-24

**Authors:** Shuaixi Yang, Yabing Yang, Yingshuai Fang, Quanbo Zhou, Weipeng Sun, Zhiyong Zhang, Weitang Yuan, Zhen Li

**Affiliations:** 1 Department of Colorectal Surgery, The First Affiliated Hospital of Zhengzhou University, Zhengzhou, China; 2 The First Clinical School of Medicine, Zhengzhou University, Zhengzhou, China

**Keywords:** Cancer therapy, Tumour immunology

## Abstract

The emergence of immunotherapy has heralded a new era in cancer treatment, with countless patients reaping the benefits of this innovative approach. While the majority of immunotherapy research has concentrated on T cells, there is a growing body of evidence highlighting the significant role of tumor-infiltrating B cells(TIL-Bs) in tumor immunity. This review synthesizes the potential mechanisms by which B cells contribute to tumor proliferation, metastasis, drug resistance, and angiogenesis. We provide a comprehensive analysis of the role of TIL-Bs within the tumor microenvironment(TME) and their impact on the cancer immune response, emphasizing their dual role as both allies and adversaries in the fight against cancer. To explain this phenomenon, we propose a dynamic regulatory framework of the TME targeting B cells, which indicates that the functions of B cells adjust in response to the dynamic changes of the tumor microenvironment. Understanding the mechanisms of B cell action within the TME is crucial for the development of targeted immunotherapies that leverage TIL-Bs. Finally, this article summarizes the latest advances in TIL-Bs in cancer immunotherapy and provides a historical overview of the evolution of immunotherapeutic strategies.

## Facts

1. The role of B cells in tumor proliferation is complex. On the one hand, B cells participate in anti-tumor immunity directly or indirectly through the secretion of antibodies, cytokines, or antigen presentation. On the other hand, B cells can promote tumor progression by suppressing immune responses or promoting an inflammatory environment.
2. B cells play an important role in tumor metastasis. B cells themselves can directly inhibit tumor cell metastasis and can also assist other immune cells in suppressing tumor metastasis. In addition, B cells can indirectly promote tumor metastasis by suppressing immune responses.
3. The levels of specific B cell phenotypes and B cell metabolites in the tumor microenvironment are associated with tumor drug resistance.
4. B cells can promote tumor angiogenesis by releasing angiogenic factors, producing antibodies, or activating specific gene pathways.
5. Immune checkpoint inhibitor therapy has shown potential in modulating B cell responses and enhancing the efficacy of cancer immunotherapy.

## Open questions

1. What are the specific mechanisms of B cells in tumor proliferation, metastasis, drug resistance, and angiogenesis? Can new therapeutic targets be developed?
2. What is the specific mechanism of B cells in immune checkpoint inhibitor therapy?
3. What are the specific roles of B cells in B cell depletion therapy, B cell-related cytokine therapy and cancer vaccines?

## Introduction

B cells, originating from hematopoietic stem cells, are the primary humoral immune cells and play a significant role in the body’s anti-tumor immunity. T cells have long been considered the main participants in anti-tumor immunity. However, in recent years, the discovery of the complex relationship between B cells and tumors has drawn attention to the study of B cells within the TME. Studies have shown that within the intricate TME, there exist various subtypes of TIL-Bs. Some B cell subtypes exert anti-tumor effects by secreting antibodies, antigen presentation, and the secretion of cytokines [1–4]. On the other hand, some B cell subtypes act as accomplices to the tumor, either directly or indirectly. Among them, regulatory B cells (Bregs) are particularly representative, exhibiting pro-tumor effects by suppressing tumor immune functions through the secretion of various cytokines [5]. The relationship between B cells and the TME is complex; TIL-Bs not only directly affect tumor cells but also indirectly influence tumor immunity by affecting the function of other immune cells within the TME, such as T cells, Tregs, NK cells, and dendritic cells (DCs) [6]. B cells modulate important biological processes such as tumor proliferation, metastasis, drug resistance, and angiogenesis through complex signaling interactions with the TME. The complex immune effects of B cells may be associated with the dynamic regulation of the TME. Furthermore, recent studies have indicated that B cells are closely related to tertiary lymphoid structures (TLSs). B cells can reshape the TME by forming TLSs, and their phenotypic and metabolic states can affect the initiation and maturation of TLSs.

Humanity has long recognized the importance of the immune system and has harnessed it to treat diseases. For instance, in ancient China, physicians used extracts from the pustules of smallpox patients to prevent the disease. With the advancement of science and technology in modern times, our understanding of the immune system has deepened. An increasing number of studies are dedicated to the research of immunotherapy. The advent of immunotherapy has provided new approaches to disease treatment, particularly for tumors, offering new hope to patients who were previously untreatable. To date, the majority of research related to tumor immunotherapy has focused on T cells. However, mounting evidence suggests that B cells hold significant potential in tumor immunotherapy [7]. For example, in soft tissue sarcomas, B cells have emerged as the strongest prognostic factor for immune therapy responses [8]. This underscores the importance of TIL-Bs in tumor progression, indicating that they may be important targets for tumor immunotherapy. Furthermore, immune checkpoint inhibitor (ICI) therapy has become a first-line treatment for various cancers, yet some patients do not benefit from ICI. Therefore, investigating the underlying mechanisms of ICI therapy is of great significance for cancer treatment. Some studies have shown that the abundance of B cells in cancer is associated with a favorable prognosis for ICI [9].

**Fig. 1:**
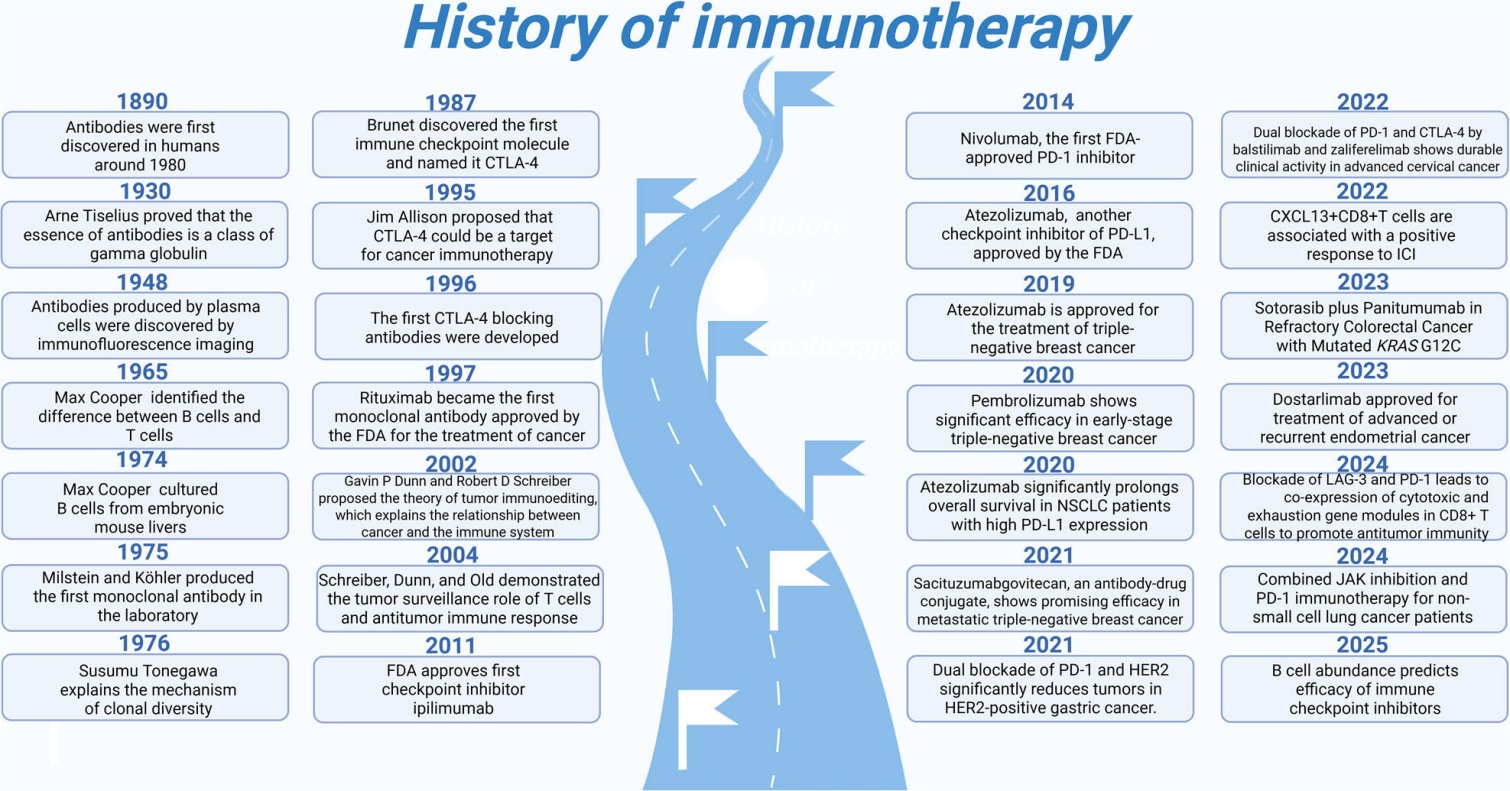
History of immunotherapy (Created with BioRender.com).

The complex network interactions between B cells and TME offer numerous potential targets for the development of targeted drugs. With the accumulation of more research evidence, B cells are expected to become a new focus in tumor immunotherapy. To facilitate these exciting research endeavors, this article summarizes the complex roles of tumor-infiltrating B cells in the TME and the close associations between B cells and TLSs. Additionally, we discuss the mechanisms of action and latest advancements in immunotherapy, and systematically review the developmental history of immunotherapy (Fig. 1).

## Development and phenotype of B cells

### Development of B cells

**Fig. 2:**
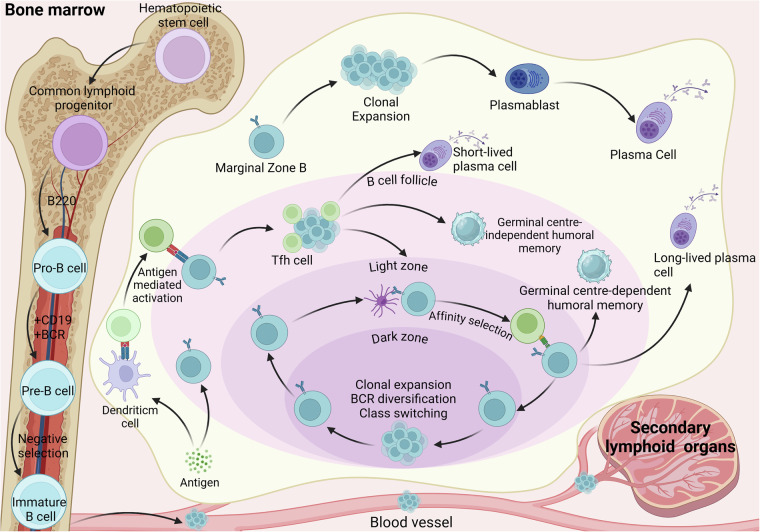
B-cell development and maturation. B cell development begins in the bone marrow, where they gradually develop into immature B cells. Before leaving the bone marrow, B cells undergo negative selection to eliminate self-reactive cells.B cells further develop and mature in secondary lymphoid organs throughout the body. Germinal center follicles are the primary site of somatic cell hypermutation and antibody class switching. After proliferation and differentiation, they eventually give rise to long-lived plasma cells or memory B cells capable of secreting highly specific antibodies. In the marginal zone, developing B cells are able to present antigens in a T-cell-independent manner, thereby giving rise to plasma cells. (Created with BioRender.com).

The ontogeny of B cells within the bone marrow is marked by two pivotal developments: the expression of functional B cell receptors (BCRs) and the acquisition of self-tolerance. The bone marrow microenvironment, notably the cytokines and adhesion molecules secreted by stromal cells, is instrumental in facilitating B cell maturation. This developmental trajectory progresses through distinct stages: pro-B, pre-B, immature B cells, and culminates in mature B cells. Central immune tolerance is established in immature B cells through a series of intricate mechanisms, including clonal deletion, receptor editing, and anergy. Upon maturation, B cells relocate to the spleen and lymph nodes to engage in peripheral immune responses. Naive B cells, upon antigenic stimulation, undergo differentiation into memory B cells or plasma cells (PCs), a process known as B cell activation. Functionally distinct B cell subsets include memory B cells, FOBs, and Bregs [10–12] (Fig. 2).

Following their egress from the bone marrow, B cells transition into the spleen, where they are designated as transitional B cells characterized by the expression of rearranged BCRs and the marker B220 [13]. Within the splenic environment, T1 B cells undergo progressive maturation, culminating in the T2 B cell stage. Subsequently, T2 B cells in the spleen diverge into distinct lineages: follicular (FO) B cells, marginal zone (MZ) B cells, and B1 cells [14]. Both FO and MZ B cells are equipped to respond to specific antigens. MZ B cells are resident in the spleen, localizing to the marginal zones where they are poised to encounter circulating antigens early on. These cells then proliferate and differentiate into PCs, secreting antibodies in response. In contrast, circulating FO B cells are found in the spleen follicles, lymph nodes, and bone marrow, and upon encountering T cell-dependent antigens, they differentiate into plasmablasts and PCs, albeit at a pace that is more protracted compared to their MZ counterparts [14, 15].

### Phenotype of B cells

B cells are classified into B-1a, B-1b, and B-2 cells based on their contributions to innate and adaptive immunity. B-1 cells, which originate from the liver during embryogenesis, are self-renewing and constitute a component of the innate immune system, acting early in immune responses. B-2 cells are the principal antibodies-secreting cells involved in humoral immunity, differentiating into PCs under antigenic stimulation and T helper cell assistance to produce antibodies [11]. B-2 cells can be further categorized into immature transitional cells (T1, T2, and T3) and mature FO B cells or MZ B cells. MZ B cells ultimately differentiate into PCs, while FOB cells give rise to short-lived PCs within 1-2 days and 3-5 days, respectively [15, 16]. Bregs, immunosuppressive cells that foster immune tolerance, inhibit the functions of T cells and other pro-inflammatory cells through the production of IL-10, IL-35, and TGF-β. Emerging evidence suggests that immunosuppression is not attributed to a specific Breg phenotype but rather represents a dynamic equilibrium among multiple B cell subsets and other immune cells. For example, B10 cells in the spleen produce IL-10 and suppress effector CD4^+^ T cells, monocytes, and DCs. Immature cells, identified in the blood and inflammatory sites, produce IL-10, induce Treg cells, suppress Th1 and Th17 cells, and inhibit virus-specific CD8^+^ T cell responses [7, 17]. Additionally, the recently identified DUSP4⁺ atypical memory (AtM) B cells exhibit an exhausted and bystander-activated phenotype. These cells are capable of secreting antibodies that recognize self-antigens and inhibiting T cell function, thereby maintaining the tumor microenvironment in an immunosuppressive state [18].

Recent studies have identified specific B cell phenotypes with significant implications in tumor immunity. IgA^+^ B cell clusters, for instance, inhibit CTL expansion and cytotoxicity through IL-10, PD-L1, and Fas ligand (FASL), promoting tumor growth [19]. In a mouse model of pancreatic cancer, IL-35^+^ Bregs with immunosuppressive functions have been identified [20]. In human melanoma, PD-L1^+^ Bregs have been shown to inhibit T cell secretion of IFN-γ in vitro [21]. In human liver cancer, Bregs expressing IL-10 and PD-1 have been demonstrated to suppress CD8^+^ T cell responses [22]. Furthermore, in a study on tumor lung metastasis, CD25^+^B220^+^CD19^+^ B cells were found to interact with CD4^+^ T cells and secrete TGF-β, inducing the conversion of CD4^+^ T cells into FoxP3-expressing Tregs and thereby promoting lung metastasis [23]. Notably, several specific B cell subsets may be associated with the prognosis of cancer treatment. A study on breast cancer revealed that an ICOSL⁺ B cell subset emerges after chemotherapy; this subset is linked to a better prognosis, and it can promote the proliferation of tumor-specific T cells while reducing regulatory T cells (Tregs) [24]. In another study, tumor-associated atypical B cells (TAABs) and stress-response memory B cells were identified. TAABs exhibit high levels of clonal expansion, strong proliferative capacity in tumor tissues, and a highly activated transcriptional state. Importantly, the characteristics of TAABs are associated with patient survival, and TAABs may serve as predictive markers for responses to immunotherapy. However, stress-response memory B cells highly express stress response-related genes, which is correlated with poor prognosis or non-response to immunotherapy in cancer patients [25].

## Complex role of B cells in tumor microenvironment

The tumor microenvironment (TME) is a complex structure composed of tumor cells, stromal cells, and endothelial cells, and the crosstalk between tumor cells and immune cells is a key characteristic of it. A large body of research has shown that immune cells in the TME play a crucial role in either controlling or promoting tumor growth. The role of T lymphocytes in the TME has been well characterized; however, the role of B cells in the TME remains unclear. Understanding the complex interactions between tumors and the TME is essential for developing novel therapeutic strategies that harness or modulate the immune system to combat cancer.

### Dual role of B cells in TME

**Fig. 3:**
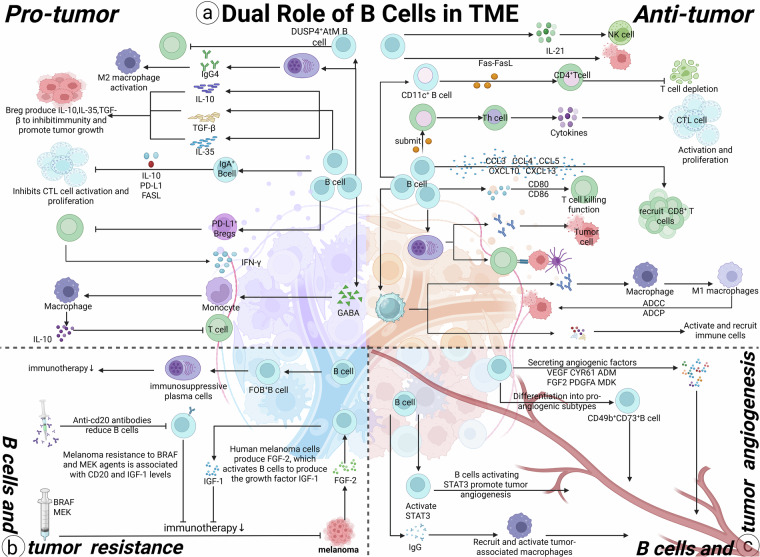
Complex role of B cells in tumor microenvironment (Created with BioRender.com). **a** B cells play a dual role in tumor proliferation and metastasis. They can inhibit tumor progression via secreting antibodies, cytokinesas well as mediating cell-cell interactions. Meanwhile, B cells are capable of promoting tumor development, predominantly throughthe suppression of anti-tumor immunity. **b** The elevated expression level of IGF-1 in B cells is associated with chemoresistance in melanoma. FOS-positive B cells correlate with poor responses to tumor immunotherapy. **c** B cells can facilitate tumor angiogenesisby expressing a variety of pro-angiogenic factors, including VEGF, CYR61, ADM, FGF2, PDGFA and MDK. The CD49b+CD73+B cell subset serves as a pro-angiogenic subset. Tumor-associated B cells with activated STAT3 can promote tumor progression by enhancing tumor angiogenesis. Additionally, B cells can indirectly stimulate tumor angiogenesis through secreting IgG and polarizing macrophages.

Numerous studies have shown that the close interaction between B cells and T cells plays a vital role in anti-tumor immunity. It is well known that B cells can directly secrete tumor-specific antibodies to kill tumor cells. Beyond this direct effect, they can also exert indirect anti-tumor activity through antibody-dependent cellular cytotoxicity (ADCC) and antibody-dependent cellular phagocytosis (ADCP) [7, 26]. An important function of B cells in the immune system is acting as APCs: they process antigens and present them via MHC class I and II molecules to CD8^+^ and CD4^+^ T cells, respectively, thereby triggering T cell-mediated immune responses. Compared with other APCs, B cells are more sensitive to antigens. On one hand, B cells help activate CD4^+^ T cells and increase T cell density in the TME. on the other hand, they drive the differentiation of CD4^+^ and CD8^+^ T cells into distinct functional subsets, enhancing T cell immune responses [27–29]. B cells can provide activation signals to T cells—such as co-stimulatory ligands CD80 and CD86—to support T cells tumor-killing function [30, 31]. Specifically, CD20^+^ B cells and CD8^+^ T cells use the CD40/CD40L co-stimulatory signal to trigger T cell-mediated tumor destruction [32] (Fig. 3a). Additionally, B cells not only activate T cells but also secrete chemokines (e.g., CCL3, CCL4, CCL5, CXCL10, CXCL13) to recruit T cells [33–35]. Memory B cells secrete CXCL8 to attract DCs to tumor metastasis sites, strengthening antigen presentation and thus promoting T cell-mediated tumor killing [36–38]. They can also secrete IL-21 to activate natural killer (NK) cells [39]. Conversely, helper T cells induce B cell differentiation into plasma cells, which then secrete tumor-specific antibodies. A recent study revealed that interactions between intratumoral CD4^+^ T cells and B cells induce the generation of tumor-specific follicular helper T (Tfh) cells; these Tfh cells then secrete IL-21 to enhance the anti-tumor effects of CD8^+^ T cells [29]. Moreover, B cells can secrete IFN-γ to promote the lysis of metastatic lesions [40] (Fig. 3a).

In addition to collaborating with T cells, B cells also engage in cooperative interactions with other cells within the TME to exert anti-tumor effects. Cancer-associated fibroblasts (CAFs) exhibit elevated expression levels of CXCL13, BAFF, and APRIL. Relying on the CXCR5-CXCL13 axis, CAFs facilitate the accumulation of B cells within the tumor, while concurrently promoting their own proliferation and the expansion of TLSs in the TME [41]. Furthermore, effector B cells can modulate the migration of DCs and mediate antibody production. These antibodies subsequently induce M1-polarized macrophages to execute ADCC or ADPC [42] (Fig. 3a).

Beyond exerting anti-tumor effects via antibody secretion, cytokine production, and intercellular crosstalk, B cells are also capable of directly eliminating tumor cells. This direct cytotoxic activity of B cells contributes to the suppression of tumor metastasis. For instance, IL-10-negative B cells exhibit elevated expression of Fas ligand (FasL) and mediate direct tumor cell killing through the Fas-FasL signaling pathway [43]. Additionally, accumulating evidence suggests that granzyme B-positive (GzmB⁺) B cells may possess cytotoxic potential [44, 45] (Fig. 3a).

While the anti-tumor role of B cells is a well-recognized fact, not all B cell subsets exert anti-tumor effects. For instance, Bregs negatively regulate immune responses by producing anti-inflammatory cytokines such as IL-10, IL-35, and TGF-β (Fig. 3a) [5]. Bregs have been detected in the peripheral blood of patients with gastric cancer, where they mediate tumor immune evasion via IL-10 signaling [46]. In cases of bone metastasis from human melanoma, programmed death-ligand 1-positive (PD-L1⁺) Bregs have been shown to inhibit interferon-γ (IFN-γ) secretion by T cells in vitro [21]. Additionally, granzyme B (GzmB) produced by Bregs can degrade the TCR on CD4⁺ T cells [47]. A recently identified subset, dual-specificity phosphatase 4-positive (DUSP4⁺) atypical memory (AtM) B cells, can suppress T cell function, leading to an immunosuppressive state within the TME [18]. Furthermore, antibodies produced by PCs may also promote tumor progression. For example, IgG4 subclass antibodies have been demonstrated to be associated with tumor malignancy and poor prognosis in patients with esophageal cancer. In another study, IgG4 was found to induce the activation of M2-polarized macrophages, which inhibits anti-tumor effects and thereby indirectly facilitates tumor development [48]. IgA may promote tumorigenesis by modulating the immune function of T cells to reduce anti-tumor activity. In a study on melanoma, the concentration of IgA was shown to correlate with poor prognosis in patients with melanoma and hepatocellular carcinoma (HCC) [49, 50]. Shalapour et al. reported that the IgA⁺ B cell population inhibits the expansion and cytotoxicity of CTLs through IL-10, PD-L1, and FasL, thereby promoting tumor growth [19]. Metabolites of Bregs also interact with the TME. One study revealed that activated B cells and PCs secrete γ-aminobutyric acid (GABA). In a subcutaneous tumor model of MC38 colorectal cancer, B cell depletion or B cell-specific inactivation of the GABA-synthesizing enzyme glutamate decarboxylase 67 (GAD67) significantly enhanced the cytotoxic function of CD8⁺ T cells. Mechanistically, GABA promotes the differentiation of monocytes into anti-inflammatory macrophages that secrete IL-10, which in turn inhibits the anti-tumor function of CD8⁺ T cells [51]. Another study identified that B cell receptor-associated protein 31 (BAP31) can promote tumor cell proliferation by stabilizing serine protease inhibitor clade E member 2 (SERPINE2), making it a potential therapeutic target for HCC [52] (Fig. 3a).

In summary, the role of B cells within the TME is highly complex. Within the TME, B cells can act not only as “killers” of tumor cells but also as “accomplices” that support tumor progression. Specifically, B cells exert anti-tumor effects through mechanisms such as antibody secretion, cytokine production, and collaboration with other immune cells; conversely, they can also promote tumor progression by suppressing the immune functions of other cells. Given the pivotal role of B cells in tumor immunity, further investigation into the crosstalk between B cells and other cellular components of the TME is essential. Such research will provide greater feasibility for the development of B cell-targeted anti-tumor therapies.

### B cells and tumor drug resistance

Drug resistance in advanced-stage cancers is a leading cause of mortality, often stemming from the complex interplay between diverse immune cells and tumor cells within the TME. Recent melanoma research has unveiled a role for tumor-associated B cells in mediating resistance to cancer therapeutics. Human melanoma cells secrete FGF-2, which in turn activates B cells to produce the growth factor IGF-1. Resistance to BRAF and MEK inhibitors in melanoma has been correlated with elevated levels of CD20 and IGF-1 transcripts in the TME, alongside increased IGF-1 expression by B cells. Clinical trial data further demonstrate that anti-CD20 antibodies exert anti-tumor effects through B cell depletion [53]. FOS^+^ B cells, identified across multiple cancer types, are linked to suboptimal responses to immunotherapy. The FOS protein can complex with JUNB to form AP-1, which promotes the expression of Blimp-1, driving B cell differentiation into immunosuppressive PCs. Moreover, FOS^+^ B cells are implicated in the modulation of tumor necrosis factor signaling pathways, hinting at mechanisms underlying their immunosuppressive activities [54]. In the context of head and neck squamous cell carcinoma (HNSCC), research has established a significant correlation between the TME and the chemosensitivity of tumors [55] (Fig. 3b).

### B cells and tumor angiogenesis

Angiogenesis plays a critical role in embryonic development, tissue growth, and wound healing, and it is equally essential for tumor growth, as tumors rely on blood supply to meet their oxygen and nutrient demands. Understanding the mechanisms underlying tumor angiogenesis is pivotal for devising novel cancer therapeutics [56]. Recent studies have revealed that B cells can stimulate tumor angiogenesis by expressing multiple pro-angiogenic factors, such as VEGF, CYR61, ADM, FGF2, PDGFA, and MDK. Moreover, a distinct pro-angiogenic subset of B cells has been characterized by the expression of CD49b and CD73 molecules [57]. In the context of melanoma, tumor-associated B cells that activate STAT3 have been shown to enhance tumor progression through the promotion of angiogenesis, implying that STAT3 in B cells could be a potential target for anti-angiogenic therapies [58]. Furthermore, B cells can indirectly stimulate tumor angiogenesis by secreting IgG and polarizing macrophages. For example, in skin cancer, IgG produced by B cells has been demonstrated to recruit and activate tumor-associated macrophages (TAMs) that foster tumorigenesis and angiogenesis, thereby promoting skin carcinogenesis [59]. These findings highlight the multifaceted role of B cells in tumor angiogenesis and suggest new avenues for therapeutic intervention(Fig. 3c).

## B cells and tertiary lymphoid structures

TLSs are ectopic aggregates formed by infiltrating immune cells, which exert anti-tumor effects analogous to those of secondary lymphoid organs [60, 61]. The presence, of distinct B-cell zones and T-cell zones is indispensable for defining TLSs. However, other cell types, such as DCs, high endothelial venules (HEVs), and fibroblasts, can also accumulate within TLSs [62]. The prognostic value of TLSs is influenced by their location, density, and maturation stage. A pioneering study categorized TLSs maturation into three stages: Early TLS (E-TLS): Characterized by T-cell and B-cell aggregates, but lacking B-cell follicles and follicular dendritic cells (FDCs). Primary follicle-like TLS (PFL-TLS): Presence of CD21^+^ FDCs within the B-cell area, with no GC responses. Secondary follicle-like TLS (SFL-TLS): Presence of a GC region containing CD21^+^CD23^+^ FDCs in the B-cell area [63]. Within TLSs, B cells can either aggregate independently or colocalize with T cells and other immune cells [64, 65]. Accumulating evidence has demonstrated that B cells in TLSs modulate the TME through multiple mechanisms, including antigen presentation, antibody production, and cytokine secretion.

**Fig. 4:**
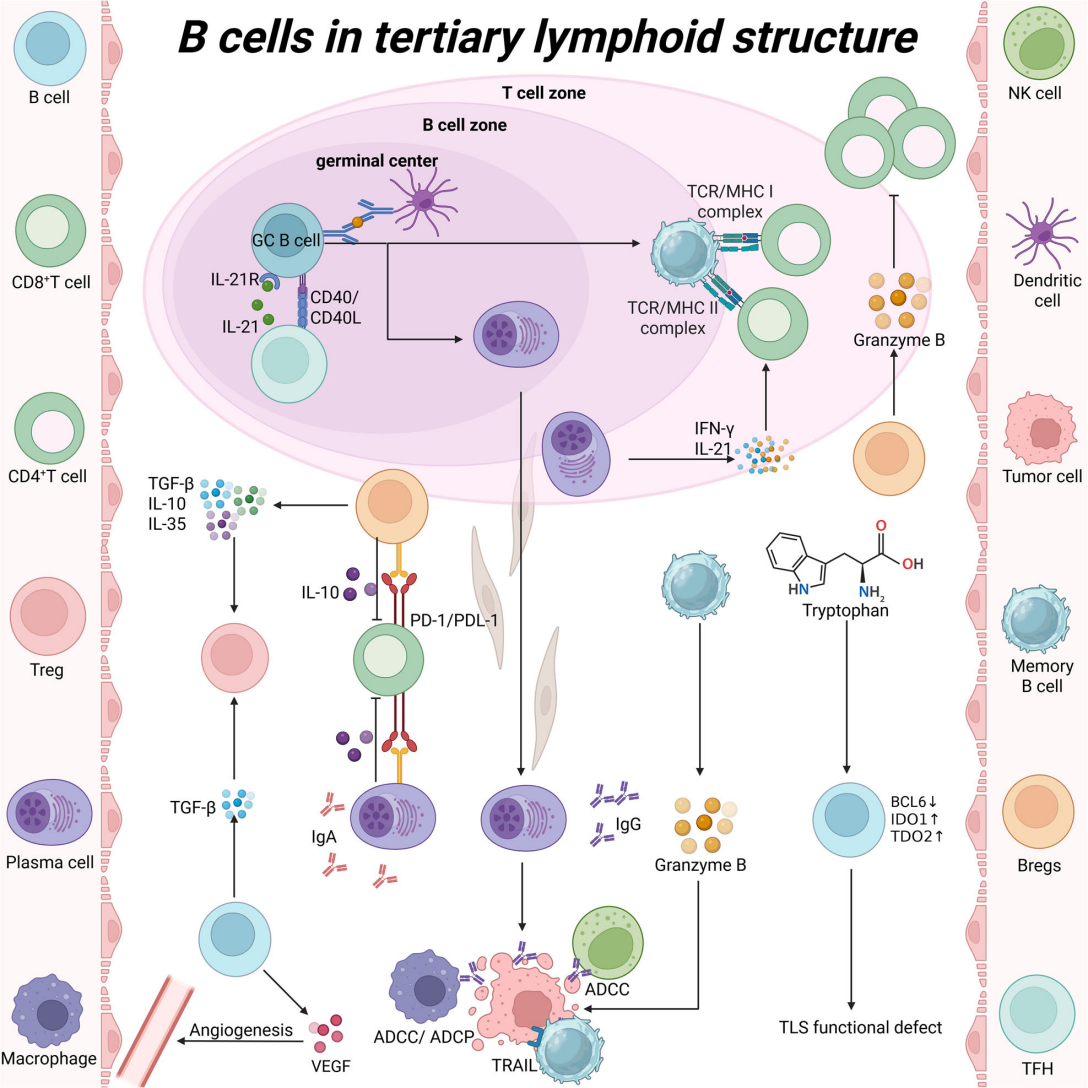
B cells in tertiary lymphoid structure. In the GC of TLSs, GC B cells receive antigen presentation from FDCs and interact with Tfh before differentiating into memory B cells and PCs. Within the T-cell compartment, B cells and DCs present antigenic peptides to effector T cells, which promotes the infiltration of effector T cells into the tumor bed. Plasma cells enter the tumor bed along the trajectory of fibroblasts and produce anti-tumor IgG antibodies. Memory B cells can kill tumor cells by expressing tumor necrosis factor-related apoptosis-inducing ligand (TRAIL) or destroy tumor cells by releasing granzyme. B Antibodies secreted by plasma cells can also eliminate tumor cells through ADCC or ADCP. IgA PCs are associated with the secretion of inhibitory cytokines, and these inhibitory cytokines create an immunosuppressive environment. B cells also produce VEGF to promote angiogenesis. Bregs induced by IL-21 secrete granzyme B, which inhibits the proliferation of CD4^+^ T cells. In deviating TLSs, the tryptophan metabolism activity of B cells is abnormally increased, the expression of BCL6 (a key germinal center transcription factor) is insufficient, while the expressions of immunosuppressive genes IDO1 and TDO2 are significantly upregulated, leading to TLS functional defects. (Created with BioRender.com).

In mature TLSs, follicular B cells are activated upon antigen encounter with T cell help, then differentiate into plasma cells or memory B cells and produce large quantities of tumor-specific antibodies to eliminate tumor cells [6]. These plasma cells are widely distributed within tumor tissues, and even a small number of them can secrete substantial amounts of antibodies. Across multiple cancer types, including renal cell carcinoma, bladder cancer, and non-small cell lung cancer, the levels of plasma cells producing IgG and IgA correlate with the prognosis of ICI therapy [41, 66, 67]. Beyond antibody secretion, these plasma cells also produce a spectrum of cytokines such as tumor TNF, IL-2, IL-6, and IFN-γ. These cytokines recruit T cells and other effector cells to TLSs, enabling tumor cell killing via cytotoxicity and phagocytosis [64] (Fig. 4). A study on nasopharyngeal carcinoma revealed that GC reactions within TLSs drive plasma cell maturation. These mature plasma cells are scattered in tumor aggregates and promote the apoptosis of Epstein-Barr virus (EBV)-associated malignant cells. Additionally. CXCL13⁺ cancer-associated fibroblasts (CAFs) enhance B cell adhesion and antibody production, while activating exhausted CXCL13⁺CD8⁺ T cells in tumor cell aggregates [68]. In a study focusing on clear cell renal cell carcinoma (ccRCC), the plasma cell-associated gene MZB1 and immunoglobulin genes IGHG1 and IGHA1 exhibited high expression levels both inside TLSs and in distant tumor regions. This finding strongly suggests that a subset of antibody-secreting plasma cells within TLSs originates from B cell activation triggered by in situ antigen stimulation and possesses the capacity to migrate to distant sites. Further trajectory analysis using specific markers for plasma cells and fibroblasts demonstrated a high degree of overlap between the migration paths of plasma cells and the distribution trails of fibroblasts, with plasma cells being encapsulated within the fibroblast network. Collectively, these lines of evidence indicate that plasma cells can achieve long-distance dissemination within the tumor bed by leveraging the “tracks” formed by fibroblasts [67] (Fig. 4).

However, not all B cells within TLSs exert pro-immunomodulatory effects. B cells have been shown to exhibit pro-tumorigenic activity by suppressing immune responses through the secretion of immunosuppressive factors, such as interleukin-10 (IL-10), interleukin-35 (IL-35), transforming growth factor-β (TGF-β), and γ-aminobutyric acid (GABA)—effects primarily mediated by Bregs [9, 69]. Notably, the definition of Bregs is not restricted to a specific B cell phenotype, as they lack lineage-specific markers. Instead, Bregs are typically identified based on the immunomodulatory factors they secrete. Bregs encompass diverse phenotypes and subsets, including B2-lineage immature transitional-2 marginal zone precursor cells (T2-MZPs), mature B1-lineage B cells in the peritoneal cavity, IL-10-producing CD5⁺CD1dhi B cells (B10 cells), and marginal zone (MZ) B cells [70]. One mechanism by which Bregs regulate anti-tumor responses involves the direct suppression of effector T cell function [71]. Lindner et al. demonstrated that IL-21-induced Bregs secrete granzyme B (GzmB). In vitro, these Bregs inhibit CD4⁺ T cell proliferation by translocating active GzmB into T cells and degrading the ζ-chain of the T cell receptor [47]. Moreover, such IL-21-induced Bregs have been detected in various solid tumors, including breast cancer, ovarian cancer, and colorectal cancer. In a study on lung adenocarcinoma, follicular helper T (Tfh)-like cells, germinal center B cells, and dysfunctional CD8⁺ T cells were found to accumulate during tumor initiation/invasion and form intra-tumoral TLSs. The anti-tumor effect of Tfh-dependent TLSs is mediated via interleukin-21 (IL-21)-IL-21 receptor signaling; inhibition of TLSs formation through depletion of Tfh cells or B cells promotes tumor growth [72] (Fig. 4).

Furthermore, the phenotypic and metabolic states of B cells may exert a critical influence on the maturation of TLSs. In a recent study, TLSs were classified into three subtypes based on the developmental trajectory of B cells: mature TLSs, conforming TLSs and deviating TLSs. Mature TLSs are enriched in GC-B cells and express key regulatory molecules such as CXCL13 and activation-induced cytidine deaminase (AICDA)—factors essential for B cell activation and antibody production. Conforming TLSs exhibit prominent T cell activation, characterized by enhanced T cell receptor (TCR) signaling, elevated expression of effector molecules, and strengthened CD40-CD40L crosstalk between Tfh cells and naive B cells. Concurrently, the upregulation of BCL6 drives B cell differentiation toward the GC lineage. In contrast, deviating TLSs display abnormally heightened tryptophan metabolic activity in B cells, insufficient expression of the key GC transcription factor BCL6, and marked upregulation of immunosuppressive genes (IDO1 and TDO2)—collectively resulting in TLS functional impairment. Notably, restricting tryptophan intake increased the proportion of mature TLSs; when combined with PD-1 inhibitors, this intervention further led to reduced tumor volume [73].

## Dynamic regulatory framework of tumor microenvironment

The TME constitutes a complex ecosystem encompassing cellular components (e.g., tumor cells, immune cells, stromal cells) and acellular components (e.g., extracellular matrix, cytokines, chemokines). It is crucial to emphasize that B cells exhibit a dual role as both “allies” and “foes” in the battle against tumors [6]. A question warranting in-depth consideration is why B cells exert opposing effects in tumor immunity. Current studies have clearly demonstrated that macrophages undergo alterations in their gene expression profiles under the influence of the TME, leading to polarization into two distinct subsets: M1-type and M2-type macrophages. M1 macrophages orchestrate anti-tumor immunity by secreting pro-inflammatory cytokines, including TNF-α and IL-12. These cytokines collectively activate cytotoxic T lymphocyte responses, induce tumor cell apoptosis, and enhance immune surveillance. In contrast, M2 macrophages are induced within the TME and promote tumor angiogenesis, immune evasion, tumor cell proliferation, and metastasis through the secretion of factors such as VEGF and TGF-β [74]. Drawing analogy to the conceptual framework of macrophage polarization, we herein propose a dynamic regulatory framework for B cells mediated by the TME. The core logic of this framework lies in the notion that the function of B cells is not fixed; instead, it adapts to the dynamic changes within the TME, ultimately resulting in the manifestation of their dual roles.

From the perspective of signaling molecules, when pro-inflammatory factors dominate the microenvironment, B cells are more prone to differentiate toward an anti-tumor phenotype and exert anti-tumor functions. For instance, one study indicated that IL-12 acts as a “cytokine switch” that acts directly on B cells, promoting their extrafollicular differentiation while inhibiting germinal center reactions. Specifically, IL-12 initiates a positive feedback loop between IL-12 and IFN-γ within B cells, amplifying IFN-γ production. This loop, in synergy with IL-12 itself, promotes the proliferation of mouse and human B cells and their differentiation into plasmablasts [75]. In contrast, when immunosuppressive factors (e.g., IL-10, TGF-β) accumulate in the microenvironment, B cells shift toward a pro-tumor phenotype. For example, IL-10 secreted by transitional B cells downregulates their own CD86 expression, which in turn inhibits T cell proliferation and the production of TNF-α [76]. TGF-β exerts a negative regulatory role at specific stages of B cell differentiation (e.g., during Ig gene rearrangement) by suppressing the initiation or transcription of Ig genes [77]. Intercellular crosstalk also plays a pivotal role. For example, CD11b⁺ myeloid cells enhance the anti-tumor activity of B cells by producing high levels of TNF-α, which promotes B cell activation and proliferation [78]. Conversely, when B cells coexist with Treg cells in the TME, Treg cells suppress the normal immune functions of B cells, inducing B cells to exert immunosuppressive effects that facilitate tumor progression [79]. Notably, the TME varies across different stages of tumor development. In the early stages of tumorigenesis, the TME is relatively “pristine,” with abundant antigenic substances and low inflammatory levels; during this period, B cells primarily exert anti-tumor effects [8, 80]. As the tumor progresses, the TME gradually becomes hypoxic, with massive accumulation of immunosuppressive molecules and abnormal buildup of metabolites. This hostile microenvironment drives functional shifts in B cells, which then switch to promoting tumor immune evasion and growth [80, 81].

## Cancer immunotherapy

At present, the role of TIL-Bs in tumors remains understudied, and there are few immunotherapies that function by inducing TIL-Bs. However, as our understanding of TIL-Bs deepens, a plethora of exciting opportunities emerge. Below, we summarize the recent advancements in this field.

### Immune checkpoint inhibitors

Throughout cancer progression, tumors develop various strategies to evade immune surveillance and counter immune responses, with immune checkpoint pathways playing a central role. ICI has revolutionized cancer treatment by inhibiting co-inhibitory signals, thereby reinvigorating antitumor immune responses and enhancing the immune system’s ability to eliminate cancer cells. The most commonly targeted pathways in ICI include CTLA-4, PD-1, and PD-L1 [82–84].

CTLA-4 is an inhibitory receptor that competes with CD28 for binding to B7-1 (CD80) and B7-2 (CD86) on antigen-presenting cells (APCs), inhibiting T cell activation [85, 86]. Anti-CTLA-4 antibodies block this interaction, extending T cell responses and promoting cancer cell elimination. This discovery led to the development of ipilimumab, the first approved ICI [87]. Following clinical trials and efficacy assessments, ipilimumab emerged as the first ICI to gain regulatory approval for cancer therapy. PD-1 exerts its function by binding to its ligands PD-L1 and PD-L2, thereby inhibiting the activation of peripheral T cells [88, 89]. This mechanism has been identified as a critical pathway for tumor immune evasion and thus represents an important therapeutic target. Additionally, PD-L1 expression has been documented across multiple tumor types and is associated with adverse prognosis in several cancers [90]. Antibodies targeting PD-1 (e.g., nivolumab, pembrolizumab) and PD-L1 (e.g., atezolizumab, avelumab, durvalumab) have demonstrated potent antitumor effects in multiple cancers, including melanoma, non-small cell lung cancer (NSCLC), renal cell carcinoma, and colorectal cancer [91–96].

Recent studies have identified PD-1^+^ and PD-L1^+^ TIL-Bs in various human malignancies [97–99], suggesting that TIL-Bs are direct targets of PD-L1 blockade. In urothelial carcinoma, the presence of stromal B cells and immature TLSs is associated with resistance to anti-CTLA-4 and anti-PD-1 combination therapy. However, mature TLSs are detected post-treatment, indicating a beneficial transformation in TIL-B responses [100]. In a lung cancer model, anti-PD-1 antibodies increased TLSs quantity and IgG production, coinciding with tumor regression [101]. Thus, ICI enhances the reactivity of both T cells and B cells against cancer, highlighting its potential to modulate the adaptive immune response within the TME (Table 1).

**Table 1:** Changes in B cells and TME after ICI treatment.

Cancer	ICI	Changes in B cells	Other biomarkers	Reference
Melanoma	Nivolumab, Ipilimumab	Higher memory B cells; Higher naive B cells	Increasing clonal BCR repertoire; Higher T cell density	[152]
melanoma	Anti-PD-1 and CTLA-4 treatment	Lower B cells; Higher CD21^lo^ B cells and plasmablasts	-	[98]
Melanoma	Nivolumab, Ipilimumab	High proportion of classs witched memory B cells, activated B cells and GC-like B cells	Increasing BCR types, Increasing B-cell RNA signatures	[153]
melanoma	Anti-PD-L1;Anti-PD-1;Anti-CTLA-4	Higher memory B cell-like score	Higher BCR abundance	[154]
NSCLC	Atezolizumab	Higher B cells; Higher plasma cells	Increasing TLSs	[41]
NSCLC	Anti-PD-1/PD-L1 treatment	Higher B cells	CD4 + /CD8 + T cell ratio decreased; Higher cytotoxic T cell	[155]
NSCLC	Anti-PD-1 treatment	CD20 + CD22 + ADAM28 + B cells in TLSs	-	[113]
NSCLC	Nivolumab	Higher IgM+ memory B cells	-	[156]
mNSCLC	PD-1/PD-L1 immune-checkpoint blockade	Lower B cells	-	[157]
RCC	Nivolumab, Ipilimumab	Higher plasma cells	IgG	[67]
RCC	Nivolumab combined with ipilimumab	Higher B cells; Higher switched memory B cells	Lower B cells in immune-related adverse events (irAEs)	[158]
m-ccRCC	nivolumab	Unswitched memory B cells enrichment	NSwM B cells correlated positively with TLS and CD20 + B cells	[159]
Cervical cancer	Anti-PD-1 treatment	Higher B cells	Higher PD-L1	[160]
HCC	Cabozantinib combined with Nivolumab	Higher B cells; Higher CD138 + B cells	Higher TNF-α	[161]
SCC (Mouse model)	Anti-PD-L1 treatment	Higher B cells	Increasing IgM, IgG	[102]
BC (Mouse model)	Anti-PD-1 and CTLA-4 treatment	Higher class-switched plasma cells	Increasing IgG	[162]
Ovarian cancer (Mouse model)	Abemaciclib	Higher B cells	Higher CXCL10 and CXCL13, Higher CD8 + T cells	[35]

### Chemotherapy and targeted therapy

Chemotherapy and targeted therapies, as traditional approaches to cancer treatment, have been instrumental in oncology for decades. Interestingly, there is a growing body of evidence suggesting that in certain chemotherapy-treated patients, the density of TIL-Bs correlates positively with treatment efficacy. The count of B cells has been proposed as a potential biomarker following chemotherapy in cancer patients [102, 103]. In a BC study, a phenotypic transition of TIL-Bs to ICOSL^+^ B cells post-chemotherapy was observed, with a positive correlation between chemotherapy response and TIL-Bs. This phenomenon was mirrored in murine models following chemotherapy [104]. In ovarian cancer patients responding to neoadjuvant chemotherapy, the density of CD20^+^ B cells was found to correlate positively with treatment outcomes [105]. These findings imply a significant association between B cell density and response to chemotherapy. The interplay between targeted drugs and TIL-Bs may vary; in melanoma, a negative correlation was observed between TIL-Bs and the efficacy of BRAF and MEK inhibitors. Furthermore, STING agonists have been shown to increase the density of TIL-Bs in melanoma and may potentially induce the formation of TLSs [106].

Therapeutic strategies that precisely target pathways implicated in the development and differentiation of TIL-Bs may offer a novel and potent approach to cancer treatment. These include the CD40/CD40L pathway, the BAFF/BAFFR pathway, B cell translocation gene 1 (BTG1), the TNF family, and microRNAs [7, 16]. CD40, a pivotal B cell surface molecule in mediating T cell responses, has been shown to enhance TIL-B density and inhibit tumor growth when targeted with antibodies in mesothelioma models [107]. In glioma models, anti-CD40 antibody treatment has induced the formation of TLSs and other lymphoid aggregates, albeit in the context of diminished T cell functionality, suppression by CD11b^+^ B cells, and attenuated responses to ICI [108]. The TNF family and its downstream signaling molecules, including BTG1 and microRNAs, are integral to the processes of B cell proliferation, differentiation, and apoptosis. Clinical trials involving drugs that target the TNF family and its ligands have produced encouraging outcomes [109]. In hepatocellular carcinoma (HCC), lower expression levels of BTG1 mRNA and protein have been correlated with significantly improved survival rates [110]. While TIL-Bs present a promising avenue in the realms of chemotherapy and targeted therapy, further investigation is warranted to fully understand their mechanisms of action and to harness their potential in cancer treatment strategies.

### Cytokine therapy

The discovery of cytokines with tumor-inhibitory effects is of paramount importance in oncology. Cytokine-driven signaling pathways, including the CXCL13/CXCR5 axis and the CCL19/21/CCR7 axis, are instrumental in the recruitment of B cells and the formation of TLSs within tumors. The CXCL13/CXCR5 axis is pivotal for the aggregation of CXCR5-expressing B cells and other immune cells within the TME and is closely correlated with TLSs expression and development. The CCL19/21/CCR7 axis facilitates the homing of immune cells to lymphoid tissues, enhancing immune infiltration within the TME [9, 69]. IL-2, IL-15, and IL-21 are known to foster the maturation and class-switch recombination of B cells [111]. BAFF and CXCL13, cytokines that typically portend a favorable prognosis, may be leveraged to enhance TIL-B responses and TLSs formation [112]. Furthermore, cytokines such as IL-17 and CXCL12 contribute to B cell maturation [113]. Bregs secrete immunosuppressive factors, including IL-10 and TGF-β. IL-10 suppresses the functionality of cytotoxic cells like CD8^+^ T cells, NK cells, and Th1 cells, while TGF-β drives the differentiation of B cells into IgA PCs. These IgA PCs secrete IL-10 and express immunomodulatory receptors such as PD-L1 and FAS-L, thereby further inhibiting cytolytic activity [5, 114].These results suggest that cytokine therapies have great potential to modulate B-cell responses and shape the TME to improve cancer treatment outcomes.

### Cancer vaccines

B cells fundamentally produce antibodies upon encountering tumor-specific antigens, targeting and neutralizing these antigens. Although B cells exhibit heightened sensitivity to antigens, they still require CD4^+^ T cell-mediated antigen-antibody interactions and assistance from MHC-II molecules [115]. This necessitates the determination of the structure and conformation of MHC-processed antigens, which serve as the basis for ex vivo processing and loading, and their application in the form of vaccines to cancer patients to induce adaptive immunity in B cells [9]. Recent studies have shown that prophylactic immunization of tumor-bearing mice with tumor antigen-pulsed CD40-activated B cells can induce protective antitumor immunity against B16, leading to reduced growth of F10 melanoma and E.G7 lymphoma [116]. In another study, activation of 4-1BBL^+^ B cells with a CD40 agonist and IFN-γ elicited potent immunity against glioblastoma [117]. Additionally, B-Cell-Epitope-Based Vaccines, which involve the use of chimeric B cell epitope receptors and T cell epitopes, include various tyrosine kinases such as VEGF, HER-1, HER-3, and IGF-1R [118]. The HER-2/neu peptide vaccine used for breast and ovarian cancer is an example of a B cell epitope-based vaccine [119]. The HER2/neu (ErbB) oncogene family plays a significant role in the growth, development, and metastasis of several tumor types, including ovarian and breast cancer, and its early expression in BC is associated with a significantly increased risk of cancer recurrence post-treatment [120].

### B cell depletion therapy

The modulation of immune responses in cancer by Bregs, through the secretion of immunosuppressive cytokines such as IL-10 and TGF-β, plays a critical role in either dampening or potentiating anti-tumor immunity. B cell depletion therapy, which targets the elimination of these immunosuppressive B cells, has demonstrated promising anti-tumor efficacy. Studies have indicated that B cell deficiency augments the infiltration of CD4^+^ and CD8^+^ T cells into the TME, thereby enhancing the responsiveness to chemotherapy in mouse tumor models [19].

Although B-cell-depleting antibodies are commonly used therapeutics for autoimmune diseases and B-cell-associated malignancies, few studies have investigated the modulation of non-B-cell tumor immune responses via B-cell targeting. Given the role of Bregs in suppressing tumor immunity, strategies designed to deplete B cells have been explored in experimental models. For example, in an early tumor lung metastasis model, B-cell depletion using anti-CD20 antibodies significantly improved survival [121, 122]. However, no significant clinical benefits were observed in several solid tumors, such as renal cell carcinoma, colorectal cancer, and melanoma [123]. Furthermore, CD20 expression has been shown to correlate with favorable prognosis in non-small cell lung cancer (NSCLC), suggesting that depletion of CD20⁺ B cells may be detrimental to lung cancer treatment [124, 125]. Subsequent studies revealed that anti-CD20 antibodies may deplete anti-tumor B cells while potentially enriching CD20^low^ Bregs, which further suppress immune responses and promote tumor growth [126]. In addition, several chemical modulators have been identified to inhibit Breg expansion by inactivating STAT3 and ERK signaling. For instance, CpG oligodeoxynucleotides (CpG-ODNs) significantly reduce the population of CD20low Bregs. Resveratrol and lipoxin A4 also inhibit Breg generation and function [127–129]. Concomitantly, these interventions lead to reduced production of IL-10 and transforming growth TGF-β, thereby further decreasing Breg-induced Treg cell expansion. Moreover, certain chemotherapeutic agents, STAT3 inhibitors, MEK inhibitors, and BTK inhibitors can also modulate Breg differentiation and function [7]. Despite the demonstrated efficacy of B-cell depletion therapy to a certain extent, the specificity of markers used for Breg identification remains insufficient to support the application of Breg-targeted strategies in cellular therapy. Specifically, anti-CD20 antibodies fail to selectively deplete Breg subsets with pro-tumorigenic activity. Therefore, further investigations into subtype-specific targets of Bregs and the development of strategies for their selective elimination are warranted [16].

## The development history of immunotherapy

While immunotherapy is widely perceived as a medical advancement emerging only in recent decades, its origins can actually be traced back to the 18th century. Edward Jenner, a rural physician in the United Kingdom, demonstrated protective immunity against smallpox through inoculation with cowpox virus—an event regarded as the inception of immunization and one that has exerted a profound impact on modern medicine. After decades of evolution, immunotherapy has been translated into clinical practice for the treatment of various diseases, exhibiting promising therapeutic efficacy. As pivotal immune cells, B cells have underpinned the rapid advancement of B cell-associated immunotherapies over the past several decades. Herein, we review cancer immunotherapy in the strict sense.

Around 1890, antibodies were first discovered in humans. These small proteins primarily function by binding to cellular antigens, labeling cancer cells, and subsequently facilitating their phagocytosis by specialized immune cells. Additionally, certain antibodies can transmit immune signals to the immune system and induce immune responses [130]. In 1930, Arne Tiselius employed electrophoresis to demonstrate that antibodies are essentially a class of γ-globulins. In 1948, the production of antibodies by plasma cells was visualized using immunofluorescence imaging. In 1965, Max Cooper and colleagues utilized irradiated chicks devoid of the bursa of Fabricius and thymus to elucidate that immune cells derived from these two organs exhibit distinct functions, thereby establishing the distinction between B cells and T cells. In 1974, Max Cooper’s team successfully cultured B cells from the livers of embryonic mice; concurrently, other research groups made similar observations using mouse bone marrow cells. These findings clarified the origin of B cells in mammals [131]. In 1976, Susumu Tonegawa revealed that B cells generate a diverse repertoire of antibodies through the assembly of three types of gene segments, explaining the mechanism underlying clonal diversity [132]. In 1975, Milstein and Köhler produced monoclonal antibodies (mAbs) for the first time in the laboratory. These antibodies can target specific antigens and be mass-produced to achieve therapeutic doses [133, 134]. Antibody-based immunotherapy advanced rapidly in the subsequent decades, culminating in 1997 with the approval of rituximab by the U.S. Food and Drug Administration (FDA) as the first monoclonal antibody for the treatment of non-Hodgkin lymphoma [134, 135] (Fig. 1).

In the current cancer treatment landscape, ICIs have emerged as antibodies of significant interest. In 1982, James Allison and his team used monoclonal antibodies to identify tumor-specific antigens in mice [136]. The following year, they identified the first T cell antigen receptor [137]. The development of ICI has since transformed the cancer treatment paradigm, with the first ipilimumab clinical trial initiated in 2000 [134] (Fig. 1).

In 1987, Brunet and his team discovered the first immune checkpoint molecule, which they named CTLA-4. In 1995, Jim Allison and his colleagues identified CTLA-4 as a significant immune checkpoint molecule and proposed it as a potential target for cancer immunotherapy [87, 138]. Subsequently, the first CTLA-4-blocking antibody was developed and underwent preclinical animal testing in 1996 [87]. The FDA approval of ipilimumab in 2011 as the first checkpoint inhibitor marked a milestone in cancer immunotherapy [134]. The subsequent approval of nivolumab in 2014, the first PD-1 inhibitor, expanded the scope of immunotherapies targeting the PD-1 pathway [134]. Additional inhibitors targeting PD-1 and its ligands PD-L1 and PD-L2 have since been approved, further expanding treatment options [134]. Atezolizumab, a PD-L1 inhibitor, was approved in 2016 for multiple cancers, including melanoma, lung, and bladder cancers, with an additional approval for triple-negative breast cancer in 2019 [134, 139]. In 2020, atezolizumab demonstrated a significant prolongation of overall survival in patients with PD-L1 high-expressing NSCLC [140] (Fig. 1).

ICI has brought hope to cancer patients. Exciting discoveries have emerged in recent years. In January 2020, a study showed that pembrolizumab benefited patients with previously treated MSI-H/dMMR noncolorectal cancer [141]. In 2020, another study found that pembrolizumab was effective in early triple-negative breast cancer [142]. In March 2023, dostarlimab was discovered to treat advanced or recurrent endometrial cancer [143]. These findings offer new options for previously untreatable late-stage cancers.

The simultaneous blockade of multiple immune-related targets has shown stronger antitumor effects. In 2021, dual blockade of PD-1 and HER2 significantly reduced tumor volume in HER2-positive gastric cancer [144]. In 2022, dual blockade of PD-1 and CTLA-4 with balstilimab and zalifrelimab showed durable clinical activity in advanced cervical cancer [145]. Moreover, combining ICIs with previous cancer therapies shows superior benefits. For example, nivolumab combined with chemotherapy outperformed chemotherapy alone in OS and PFS [146]. In 2023, pembrolizumab combined with gemcitabine and cisplatin emerged as a new treatment for metastatic or unresectable biliary tract cancer [147]. In 2024, adjuvant mRNA-4157 plus pembrolizumab prolonged recurrence-free survival compared to pembrolizumab alone in high-risk melanoma patients [148] (Fig. 1).

Sacituzumab govitecan, an antibody-drug conjugate targeting Trop-2, significantly improved progression-free and overall survival in metastatic triple-negative breast cancer compared to single-agent chemotherapy [149]. In 2024, studies found that JAK inhibition could enhance checkpoint blockade immunotherapy in Hodgkin’s lymphoma [150] and benefit patients with NSCLC when combined with PD-1 immunotherapy [151].

## Conclusion

The emergence of immunotherapy has markedly expanded the therapeutic landscape for cancer patients. An increasing corpus of evidence underscores the pivotal role of immune cells in oncogenesis, tumor progression, and response to immunotherapy. B cells, once considered marginal to tumor immunity, are now under the spotlight. This review discusses the multifaceted engagement of various B cell subsets within the TME, with some subsets contributing to antitumor immunity through antibody secretion, antigen presentation, and cytokine production, thus impeding tumorigenesis. The prognosis of certain cancer patients is intricately linked to immune cell infiltration, a correlation we substantiate by examining various cancer types. Patients exhibiting positive responses to immunotherapy often show heightened B cell density and activity, positioning B cells as favorable prognostic indicators in cancer. Conversely, other B cell subsets may abet cancer development and progression, influencing tumor biology and immunosuppression. The intricate biology of B cells and their dual roles in the TME necessitate further investigation to clarify their specific functions across different cancer types. Deciphering these dynamics is essential for leveraging B cells in cancer therapy and for crafting more targeted and potent immunotherapeutic strategies.

The complexity of B cell functions presents new therapeutic vistas in cancer treatment. We explore various therapeutic modalities targeting B cells, including ICIs that demonstrate potential in modulating B cell responses and bolstering the efficacy of cancer immunotherapies. Additionally, B cell depletion therapy, cytokine-based interventions, and cancer vaccines are paving new avenues for B cell-targeted immunotherapy.

In conclusion, this review underscores the imperative of a profound understanding of B cells’ role in cancer immunotherapy and the mechanisms underpinning their actions. As research continues to unveil the sophisticated roles of B cells within the TME, they are poised to become a cornerstone of next-generation cancer immunotherapies. Targeting TIL-Bs presents a promising approach to augment the efficacy of immunotherapies and to enhance patient prognoses.
